# Obituary

**Published:** 1884-11

**Authors:** 


### Obituary.

DIED.—October 16, of capillary bronchitis, Cora, aged one year and seven months, infant daughter of Dr. E. G. and Cora B. Betty, and twin sister to Lida, deceased April 30th, aged one year and two months. This is the second time within the past five months that we have been called upon to extend our sympathy to Dr. Betty and wife, and we trust it is the last bereavement we shall have to chronicle in that estimable family.

